# Structure-Based Drug Design of Small Molecule Peptide Deformylase Inhibitors to Treat Cancer

**DOI:** 10.3390/molecules21040396

**Published:** 2016-03-23

**Authors:** Jian Gao, Tao Wang, Shengzhi Qiu, Yasheng Zhu, Li Liang, Youguang Zheng

**Affiliations:** 1 Jiangsu Key Laboratory of New Drug Research and Clinical Pharmacy, Xuzhou Medical College, Xuzhou 221004, Jiangsu, China; lswangtao@163.com (T.W.); neoevens@hotmail.com (S.Q.); 18151865223@163.com (Y.Z.); 18852143485@163.com (L.L.); zhengyouguang@xzmc.edu.cn (Y.Z.); 2 Jiangsu Center for the Collaboration and Innovation of Cancer Biotherapy, Xuzhou Medical College, Xuzhou 221004, Jiangsu, China

**Keywords:** high-throughput virtual screening, human peptide deformylase, vanillin *N*-hydroxyacetamide, anticancer

## Abstract

Human peptide deformylase (HsPDF) is an important target for anticancer drug discovery. In view of the limited HsPDF, inhibitors were reported, and high-throughput virtual screening (HTVS) studies based on HsPDF for developing new PDF inhibitors remain to be reported. We reported here on diverse small molecule inhibitors with excellent anticancer activities designed based on HTVS and molecular docking studies using the crystal structure of HsPDF. The compound M7594_0037 exhibited potent anticancer activities against HeLa, A549 and MCF-7 cell lines with IC_50s_ of 35.26, 29.63 and 24.63 μM, respectively. Molecular docking studies suggested that M7594_0037 and its three derivatives could interact with HsPDF by several conserved hydrogen bonds. Moreover, the pharmacokinetic and toxicity properties of M7594_0037 and its derivatives were predicted using the OSIRIS property explorer. Thus, M7594_0037 and its derivatives might represent a promising scaffold for the further development of novel anticancer drugs.

## 1. Introduction

**Figure 1:**
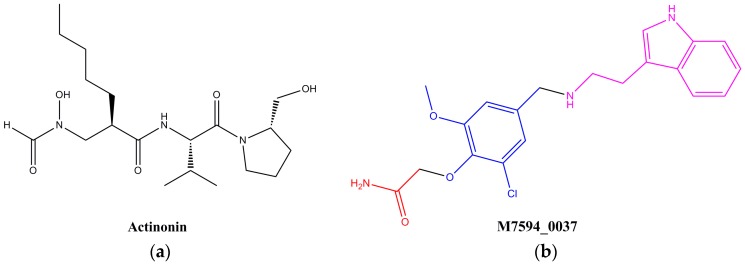
Structures of PDF inhibitor actinonin (**a**) and the shortlisted compound **M7594_0037** (**b**).

Peptide deformylase (PDF), a metalloenzyme containing Fe^2+^, is responsible for the removal of the *N*-formyl group from the terminal methionine residue in nascent proteins, which is essential to the synthesis of proteins [1,2]. In the past few decades, peptide deformylase of pathogenic microorganisms has been considered as a potential target for antibacterial drug discovery, and plenty of PDF inhibitors have been widely reported [3,4,5,6,7]. However, recent studies have demonstrated that peptide deformylase also can be found in most eukaryotes, including parasites, plants, and mammals [8,9,10]. Human genes of peptide deformylase (*def*) have been cloned and expressed, and the resulting human peptide deformylase (HsPDF) has been studied extensively [1,11,12,13]. HsPDF distributes over the mitochondrion in human cells, and also functions similarly to the PDF in pathogenic microorganisms, which catalyzes the deformylation of polypeptides [14,15,16,17]. Inhibition of HsPDF results in mitochondrial membrane depolarization and promotes cell death [13,18]. HsPDF is over-expressed in breast cancer, colon cancer and lung cancer, and the inhibition of HsPDF would significantly reduce the proliferation of these cancer cells [19]. Actinonin (Figure 1), the first found naturally occurring bacterial PDF inhibitor, also shows strong HsPDF inhibitory activity [11,20]. As suggested above, HsPDF is a promising novel target for the development of anticancer drugs [12,21,22,23,24,25].

So far, reports on inhibitors of HsPDF still remain to be conducted. The few inhibitors at present include a class of peptide inhibitors based on actinonin reported by Mona’s research group [22] and two series of non-peptide inhibitors reported by Christophe’s research group [23,24]. In addition, in view of the fact that the crystal structure of HsPDF has been reported, high-throughput virtual screening (HTVS) based on HsPDF remains to be reported. Hence, this study was intended to discover novel small molecule inhibitors with novel structural class based on our drug design strategies involving high-throughput virtual screening and molecular docking techniques. The resulting new structure was named **M7594_0037**, and the bioactivity evaluation substantiated its superior anticancer activity. To optimize the structure of **M7594_0037**, our research group designed three derivatives of vanillin *N*-hydroxyacetamide. One of them, **NA-2**, was observed to have better anticancer activities against MCF7 and HeLa cell lines compared to **M7594_0037**. Molecular docking studies also indicated that the derivatives of vanillin *N*-hydroxyacetamide could have a stable interaction with HsPDF, which was similar to the binding mode of actinonin. In summary, the derivatives of vanillin *N*-hydroxyacetamide designed in this study have the potential to become a new class of anticancer drugs.

## 2. Results and Discussion

### 2.1. High-Throughput Virtual Screening

As described in the Materials and Methods section, the crystal structure (3G5K) was utilized to screen the HsPDF inhibitors from the Topscience ChemDiv database (commercially available). The top-ranked 100 compounds were shortlisted based on the docking score and hydrogen bond. Finally, six commercially available compounds were selected from the top-ranked list of 100 by clustering analysis and were tested experimentally. Among them, **M7594_0037** (Figure 1) exhibited dramatic anticancer activities against HeLa, A549 and MCF-7 human cancer cell lines, and was selected as a lead compound for further study. The structure of **M7594_0037** differed from the common peptidic inhibitors, and it can be classified as a non-peptidic inhibitor. Non-peptidic inhibitors of HsPDF are superior to peptidic inhibitors in terms of pharmacokinetics [25].

### 2.2. Design and Synthesis of Derivatives of ***M7594_0037***

**Scheme 1:**
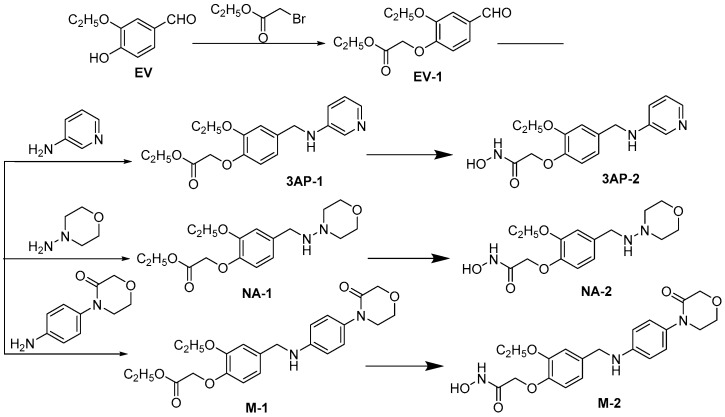
Synthesis of **M7594_0037** derivatives.

**M7594_0037** consisted of the amide at its terminal, the methyl vanillin at its middle section and the indole at its other terminal. The amide group was responsible for binding with Fe^2+^ as a metal chelating group, while the known strong metal chelating groups reported in previous papers are *N*-formyl-*N*-hydroxylamine and hydroxamic acid [25]. In other words, the amide functioning as the metal chelating group at the terminal of **M7594_0037** seemed to be a weaker one, so alteration of this group will be focused on in this article. The terminal indole ring of **M7594_0037** interacted with the hydrophobic S3 region (described in the section on molecular docking studies) where it can provide additional binding energy, selectivity, and favorable pharmacokinetic and toxicity properties [25]. Moreover, the S3 regions of the binding sites of HsPDF are generally solvent accessible, and especially large in size to accommodate various substituent groups [21]. For the purpose of improving the anticancer activity, three derivatives of **M7594_0037**, which were named **3AP-2**, **NA-2**, **M-2**, were designed. The structures of all the derivatives were characterized by ^1^H-NMR and mass spectra. The synthetic routes are shown in Scheme 1.

### 2.3. Anticancer Activity of ***M7594_0037*** and Its Derivatives

**Figure 2:**
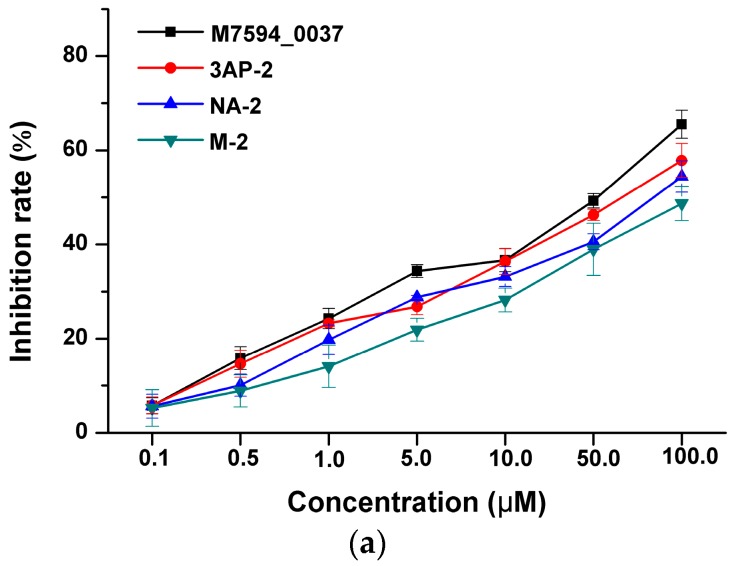

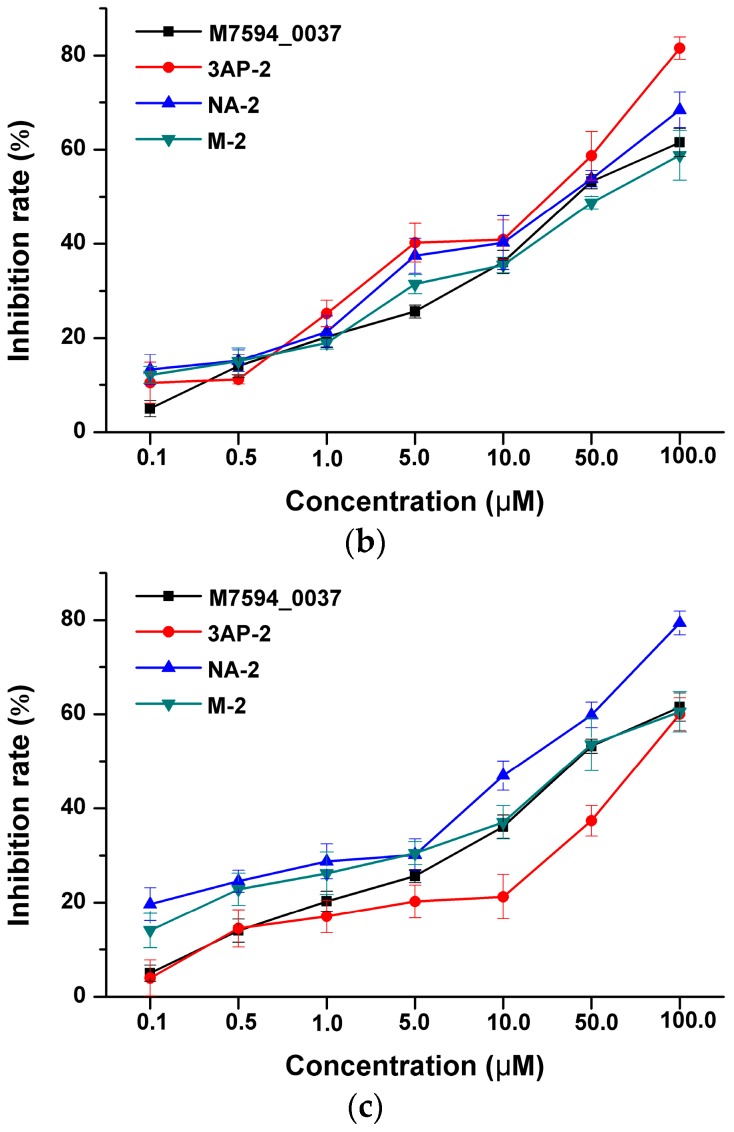
Inhibition rate of **M7594_0037** and analogs against A549 (**a**); MCF7 (**b**) and HeLa (**c**) in dose-dependence.

As we know that HsPDF has been reported to be over-expressed in many cancers, it was proven that HsPDF inhibitors could specifically be used in cancer treatment. Hence, the anticancer activities of **M7594_0037** and its derivatives were studied on HeLa, A549 and MCF-7 human cancer cell lines in a dose-dependent manner (Figure 2). The cytotoxic activity was expressed as the mean IC_50_ of three independent experiments, and the results were represented in Table 1. It is clearly shown that **M7594_0037** had inhibitory activity against HeLa, A549 and MCF-7, whose IC_50_ values are 35.26 ± 3.17, 29.49 ± 2.09 and 24.63 ± 2.19 μM, respectively. Its inhibitory activity against HeLa was slightly smaller than that of actinonin [22] (IC_50_ = 27.40 μM). For further optimization of **M7594_0037**, the amide at its terminal was replaced with *N*-formyl-*N*-hydroxylamine to give three derivatives. Each of them exhibited obvious anticancer activities. The compound **3AP-2** showed better inhibitory activity against MCF-7 than **M7594_0037** (IC_50_ = 13.62 ± 3.42 μM), while **NA-2** was even superior to **M7594_0037** in inhibition against both HeLa and MCF-7 cell lines (IC_50_ = 9.60 ± 3.04 and 22.89 ± 3.41 μM, respectively). Thus, **M7594_0037** and its derivatives have potential to become lead compounds of a class of novel anticancer drugs.

### 2.4. Binding Modes of ***M7594_0037*** and Its Derivatives

**Figure 3:**
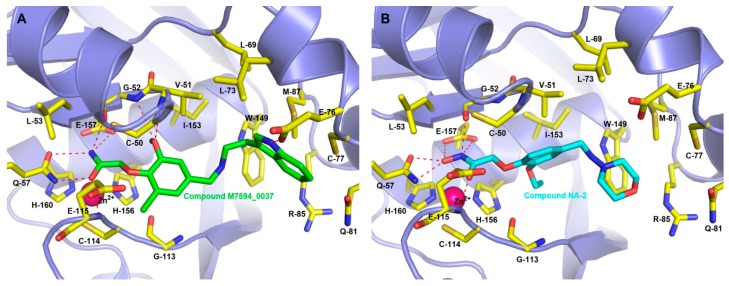
Depicting the binding pose of inhibitors in the HsPDF binding pocket: **M7594_0037** (**a**) and NA-2 (**b**). The **red** color bonds indicate the hydrogen bonds between inhibitor and amino acids (colored by yellow).

M7594_0037 and its derivatives were docked into the binding site where the ligand actinonin was placed by the Surflex module in SYBYL 7.1. The binding modes of **M7594_0037** and its derivatives with HsPDF were obtained from the results of Surflex molecular docking. As is vividly shown in Figure 3, many hydrogen bonds were formed between **M7594_0037** and HsPDF, and most of them were allowed by the amide group at the terminal of **M7594_0037**. Three hydrogen bonds were networked with side-chains of Gln-57 and Glu-157, and another three hydrogen bonds were networked with backbones of Gly-52, Glu-115. The methoxyl group at the vanillin section allowed two hydrogen bonds networked with Gly-52 and Val-51 as well, and the NH group on the indole ring allowed the hydrogen bond networked with Glu-76 to be formed. These hydrogen bonds played an important role in the binding interaction between **M7594_0037** and HsPDF. The terminal indole ring was located in the hydrophobic pocket constituted by Trp-149, Leu-73, Met-87, Arg-85, Cys-77. Analogous to the binding mode of **M7594_0037**, **NA-2** also formed stabilized hydrogen bonds with Gln-57, Gly-52, Glu-115, and Glu-157, and its terminal morpholine was also placed in a similar hydrophobic pocket. In the interaction between stabilized hydrogen bonds and **M7594_0037**, **NA-2** coincided with the conservative hydrogen bond interactions which has been reported in the past [21]. For example, actinonin would emerge from a conservative interaction between Val-51 and Gly-52, and it would be located in the parallel hydrophobic pocket as well [21].

### 2.5. In Silico Pharmacokinetic and Toxicity Predictions of ***M7594_0037*** and Its Derivatives

The *in silico* pharmacokinetic and toxicity predictions of **M7594_0037** and its derivatives were predicted using the OSIRIS property explorer. Results of the toxicity prediction study (Table 2) suggested that **M7594_0037** and **3AP-2** have no risk of toxicity, while NA-2 has a low risk of tumorigenicity and irritant effects, and **M-2** has a low risk of irritant and reproductive effects. Results of the pharmacokinetic prediction (Table 2) revealed that all compounds are good with the properties of solubility (logS), partition coefficient (clogP), drug likeness and molecular weight. The drug score was calculated in view of the contribution of logS, clogP, drug likeness, molecular weight and toxicity risk prediction parameters (Table 3). The drug score showed that **M7594_0037** and **3AP-2** have a higher drug score than **NA-2** and **M-2**. Nevertheless **M7594_0037** can be regarded as a lead compound for further designing new molecules with better pharmacokinetic properties.

## 3. Materials and Methods

### 3.1. High-Throughput Virtual Screening

The crystal structure of the HsPDF complex with actinonin (PDB ID: 3G5K [21]) was obtained from the Protein Data Bank (PDB) [26]. On account of its tetramer structure, only the A chain was adopted. The small molecule database was the ChemDiv database (commercially available) from Topscience Co. (Shanghai, China) Surflex-Dock module in SYBYL 7.1 [27] was used for high-throughput virtual screening. The space where ligand actinonin was placed was selected as the active pocket, and all water molecules were removed. To accelerate virtual screening, the maximum quantity of conformations was reduced from 20 to 10, and the maximum quantity of rotatable bonds was decreased from 100 to 50. Additionally, the default optimization of molecules before and after the docking was canceled. Other parameters were kept as default values.

### 3.2. Chemistry

#### 3.2.1. Synthesis of Ethyl 2-(2-Ethoxy-4-formylphenoxy)acetate (**EV-1**)

**EV-1** was prepared from ethyl vanillin according to a literature procedure [28]. Ethyl vanillin (5000 mg, 30.12 mmol) was mixed with 50 mL *N*,*N*-dimethyl sulfoxide and anhydrous potassium carbonate (8313 mg, 60.24 mmol). The mixture was fully stirred and added ethyl bromoacetate (7545 mg, 45.18 mmol). Then the mixture was stirred for 5 h at 50 °C. To remove the trace impurities, the reaction liquid was added distilled water and extracted with ethyl acetate for three times. Ethyl acetate was dried with magnesium sulfate anhydrous to give the white crystal EV-1. White crystals; mp: 142–143 °C; 86% yield. ^1^H-NMR (400 MHz, DMSO): δ 9.85 (s, 1H), 7.51 (d, *J* = 2.0 Hz, 1H), 7.49 (s, 1H), 7.07 (d, *J* = 8.4 Hz, 1H), 4.94 (s, 2H), 4.18 (q, *J* = 7.2 Hz, 2H), 4.12 (q, *J* = 7.2 Hz, 2H), 1.37 (t, *J* = 7.2 Hz, 3H), 1.23 (t, *J* = 7.2 Hz, 3H). MS (ESI) *m*/*z* = 253 [M + 1]^+^.

#### 3.2.2. Synthesis of Ethyl 2-(2-Ethoxy-4-((pyridin-3-ylamino)methyl)phenoxy)acetate (**3AP-1)**

**3AP-1** was prepared from **EV-1** according to a literature procedure [29]. EV-1 (500 mg, 1.98 mmol) and 3-aminopyridine (205 mg, 2.18 mmol) were dissolved in 10 mL 1,2-dibromoethane, and the solution was added sodium triacetoxyborohydride (630 mg, 2.97 mmol) and stirred for 2 h at the room temperature in nitrogen atmosphere. The reaction was quenched with saturated sodium bicarbonate. The reaction liquid was extracted with ethyl acetate for three times, and the ethyl acetate was dried with magnesium sulfate anhydrous and concentrated by reducing pressure to obtain the white solid, which was separated by Silica Gel Column Chromatography to give **3AP-1**. White solid; mp: 307–308 °C; 77% yield. ^1^H-NMR (400 MHz, DMSO): δ 7.97 (s, 1H), 7.74 (d, *J* = 4.4 Hz, 1H), 7.05–7.00 (m, 2H), 6.90–6.79 (m, 3H), 6.40 (s, 1H), 4.72 (s, 2H), 4.18 (q, *J* = 6.0 Hz, 4H), 4.02 (s, 2H), 1.31 (t, *J* = 7.2 Hz, 3H), 1.20 (t, *J* = 7.2 Hz, 3H). MS (ESI) *m*/*z* = 331 [M + 1]^+^.

#### 3.2.3. Synthesis of 2-(2-Ethoxy-4-((pyridin-3-ylamino)methyl)phenoxy)-*N*-hydroxyacetamide (**3AP-2**)

**3AP-2** was prepared from **EV-1** according to a literature procedure [30]. Sodium (101 mg, 4.4 mmol) was dissolved in 3 mL absolute methanol, and hydroxylamine hydrochloride (140 mg, 2.0 mmol) was dissolved in 5 mL absolute methanol. The mixture was stirred at room temperature for 10 min, and then filtered to obtain the white solid, which was flushed with absolute methanol. 3AP-1 (330 mg, 1.0 mmol) was mixed to the filtrate. The mixture was refluxed at 70 °C for 2 h and then concentrated by reducing pressure to obtain residues. Residues were dissolved with least amount of distilled water, and acidified with acetic acid to pH 4 to present the yellow sediment, which can be filtered and purified by Silica Gel Column Chromatography to give **3AP-2**. Yellow oil; 54% yield. ^1^H-NMR (400 MHz, DMSO): δ 7.97 (s, 1H), 7.73 (d, *J* = 4.40 Hz, 1H), 7.05–7.01 (m, 2H), 6.91–6.84 (m, 3H), 6.40 (s, 1H), 4.40 (s, 2H), 4.20 (d, *J* = 6.0 Hz, 2H), 4.01 (q, *J* = 7.2 Hz, 2H), 1.32 (t, *J* = 7.2 Hz, 3H). MS (ESI) *m*/*z* = 318 [M + 1]^+^.

#### 3.2.4. Synthesis of Ethyl 2-(2-Ethoxy-4-((morpholinoamino)methyl)phenoxy)acetate (**NA-1**)

**NA-1** was prepared from **EV-1** according to a literature procedure [29]. EV-1 (500 mg, 1.98 mmol), 3-aminopyridine (222 mg, 2.18 mmol), 1,2-dibromoethane (10 mL), sodium triacetoxyborohydride (630 mg, 2.97 mmol). NA-1, white solid; mp: 272–273 °C; 81% yield. ^1^H-NMR (400 MHz, DMSO): δ 7.63 (s, 1H), 7.23 (s, 1H), 7.04 (d, *J* = 8.4 Hz, 1H), 6.85 (d, *J* = 8.4 Hz, 1H), 4.77 (s, 2H), 4.17 (q, *J* = 7.2 Hz, 2H), 4.05 (q, *J* = 7.2 Hz, 2H), 3.75 (t, *J* = 4.8 Hz, 4H), 3.06 (t, *J* = 4.8 Hz, 4H), 1.35 (t, *J* = 6.8 Hz, 3H), 1.23(t, *J* = 6.8 Hz, 3H). MS (ESI) *m*/*z* = 337 [M − 1]^+^.

#### 3.2.5. Synthesis of 2-(2-Ethoxy-4-((morpholinoamino)methyl)phenoxy)-*N*-hydroxyacetamide (**NA-2**)

**NA-2** was prepared from **NA-1** according to a literature procedure [30]. Sodium (101 mg, 4.4 mmol), absolute methanol (3 mL), hydroxylamine hydrochloride (140 mg, 2.0 mmol), NA-1 (338 mg, 1.0 mmol). NA-2, yellow oil; 60% yield. ^1^H-NMR (400 MHz, DMSO): δ 7.63 (s, 1H), 7.22 (s, 1H), 7.05 (d, *J* = 7.6 Hz, 1H), 6.95 (d, *J* = 8.4 Hz, 1H), 4.41 (s, 2H), 4.04 (q, *J* = 6.8 Hz, 2H), 3.74 (t, *J* = 4.4 Hz, 4H), 3.05 (t, *J* = 4.4 Hz, 4H), 1.35 (t, *J* = 7.2 Hz, 3H). MS (ESI) *m*/*z* = 324 [M − 1]^+^.

#### 3.2.6. Synthesis of ethyl 2-(2-Ethoxy-4-(((4-(3-oxomorpholino)phenyl)amino)methyl)phenoxy)acetate (**M-1**)

**M-1** was prepared from **EV-1** according to a literature procedure [29]. EV-1 (500 mg, 1.98 mmol), 4-(4-aminogen-yl)-3-morpHolinone (419 mg, 2.18 mmol), 1,2-dibromoethane(10 mL), sodium triacetoxyborohydride (630 mg, 2.97 mmol). M-1, yellow oil; 75% yield. ^1^H-NMR (400 MHz, DMSO) : δ 7.01–6.99 (m,3H), 6.83–6.79 (m, 2H), 6.57 (d, *J* = 6.8 Hz, 2H), 6.27 (s, 1H), 4.72 (s, 2H), 4.18–4.13 (m, 6H), 4.03 (q, *J* = 7.2 Hz, 2H), 3.93–3.90 (m, 2H), 3.58 (t, *J* = 4.8 Hz, 2H), 1.32 (t, *J* = 7.2 Hz, 3H), 1.21 (t, *J* = 7.2 Hz, 3H). MS (ESI) *m*/*z* = 429 [M + 1]^+^.

#### 3.2.7. Synthesis of 2-(2-Ethoxy-4-(((4-(3-oxomorpholino)phenyl)amino)methyl)phenoxy)*-N-*hydroxyacetamide (**M-2**)

**M-2** was prepared from **M-1** according to a literature procedure [30]. Sodium (101 mg, 4.4 mmol), absolute methanol (3 mL), hydroxylamine hydrochloride (140 mg, 2.0 mmol), M-1 (415 mg, 1.0 mmol). M-2, yellow oil; 67% yield. ^1^H-NMR (400 MHz, DMSO): δ 10.65 (s, 1H), 8.95 (s, 1H), 7.01–6.99 (m, 3H), 6.9–6.84 (m, 2H), 6.57 (d, *J* = 8.8 Hz, 2H), 6.25 (s, 1H), 4.40(s, 2H), 4.18 (d, *J* = 6.0 Hz, 2H), 4.13 (s, 2H), 4.02 (q, *J* = 7.2 Hz, 2H), 3.92 (t, *J* = 4.8 Hz, 2H), 3.58 (t, *J* = 4.8 Hz, 2H), 1.33 (t, *J* = 6.8 Hz, 3H). MS (ESI) *m*/*z* = 416 [M + 1]^+^.

### 3.3. Anticancer Activity Evaluation of ***M7594_0037*** and Its Derivatives

The compounds to be measured were made in solutions with the concentrations of 1.25 μg/mL, 2.5 μg/mL, 5 μg/mL, 10 μg/mL, 15 μg/mL and were tested with HeLa, A549 and MCF-7 cell lines, respectively. The three cell lines were seeded with the density of 5000/mL on 96-well plates. Three to five duplicate wells were reserved for every concentration and one well was kept blank for control group. All these 96-well plates were kept in incubator which was at 37 °C and with an atmosphere of 5% CO_2_ for 24 h. Compounds with different concentration were added, functioning for 24 h, 48 h or 72 h. Sulforhodamine B (SRB) was adopted to detect the inhibitory rates of PDT against cells, as described in the following procedure. Culture solution was absorbed after compounds functioning for 24 h, 48 h, 72 h, then 100 µL pre-cooling 10% TCA was added to every well for fixation. The 96-well plates were then placed in refrigerator for 1 h. Fixative solution was dumped, and plates were washed and spun dry. Then 100 µL 0.4% SRB was added to every well, and use the 1% glacial acetic acid to scour off the free dyes. All plates were spun dry and left until dry as the pervious step. Then 150 µL 10 mM tris was added into every well accurately, and put the plates in shaker for 5 to 10 min to dissolve all dyes. Finally, optical delnsity (OD) values were determined with ELIASA at wavelength of 564 nm, and subtract the blank OD value when measuring. Data processing: inhibition ratio = (mean OD value of control group—mean OD value of each group)/mean OD value of control group. Use the SPSS 11.5 to calculate IC_50_.

### 3.4. Molecular Docking

For the sake of exploring the binding mode between derivatives of vanillin *N*-hydroxyacetamide and HsPDF, Surflex module in SYBYL 7.1 [27] was used for molecular docking. Parameters basically stay the same as the virtual screening, and only the maximum quantity of conformations and the maximum quantity of rotatable bonds were set to the default values: 20 and 100. Meanwhile molecular optimization before and after the docking was activated.

### 3.5. In Silico Pharmacokinetic and Toxicity Prediction of ***M7594_0037*** and Its Derivatives

The pharmacokinetic and toxicity prediction of **M7594_0037** and its derivatives were predicted using OSIRIS property explorer which uses Chou and Jurs algorithm, based on computed atom contributions [31]. Factors of toxicity risk management composed of mutagenicity, tumorigenicity, irritant and reproductive effects, relied on a precomputed set of structural fragment that give rise to toxicity alerts in case they are encountered in the studied structures [32]. These fragment were created by rigorously shredding all compounds of the RTECS database known to be active in a certain toxicity class (e.g., mutagenicity). Pharmacokinetic prediction was comprised of solubility (logS), partition coefficient (clogP), drug likeness and molecular weight properties [32]. The drug score was calculated by combining with drug likeness, cLogP, logS, molecular weight and toxicity risks in one handy value than may be used to judge the compound's overall potential to qualify for a drug.

## 4. Conclusions

PDF has been regarded as a double target for antibacterial and antitumor drugs. The research on PDF as an antibacterial target was launched previously and a considerable sum of small molecular compounds have come into clinical trials. In contrast, research on an anticancer target based on HsPDF started later, and a potent HsPDF inhibitor still remains to be found.

In view of the lack of reports on HsPDF inhibitors and the fact that virtual screening based on the HsPDF receptor protein remains to be reported, we take advantage of virtual screening to obtain the small molecular compound **M7594_0037** with an all-new structure. It has been validated that it had obvious inhibitory activity against HeLa, A549 and MCF-7 human cell lines. For the optimization of **M7594_0037**, three vanillin *N*-hydroxyacetamide analogs were designed and synthesized. Among them, NA-2 had better anticancer activity than **M7594_0037**. Moreover, we investigated the binding modes of **M7594_0037** and its derivatives with HsPDF. In the end, we evaluated the pharmacokinetic and toxicity properties of **M7594_0037** and its derivatives. Therefore, the derivatives of vanillin *N*-hydroxyacetamide have great potential to become a class of novel anticancer compounds with high activity.

## Figures and Tables

**Table 1:** Effect (IC_50_) of **M7594_0037** and analogs on human cancer cell lines.

Compounds	A549 (μM)	MCF7 (μM)	HeLa (μM)
**M7594_0037**	29.49 ± 2.09	24.63 ± 2.19	35.26 ± 3.17
**3AP-2**	45.37 ± 2.08	13.62 ± 3.42	96.57 ± 3.73
**NA-2**	67.85 ± 2.22	22.89 ±3.41	9.60 ± 3.04
**M-2**	119.95 ± 3.71	48.02 ± 2.32	36.39 ± 3.90

**Table 2:** *In silico* pharmacokinetic and toxicity prediction of **M7594_0037** and its derivatives.

Parameters	M7594_0037	3AP-2	NA-2	M-2
**Toxicity risk management**				
(a) Mutagenicity ^a^	No	No	No	Yes
(b) Tumorigenicity ^b^	No	No	Yes	Yes
(c) Irritant ^c^	No	No	Yes	No
(d) Reproductive effective ^d^	No	No	No	No
**Solubility ^e^**	−4.08	−2.59	−1.68	−3.47
**cLogP**	2.1	0.61	−1.29	0.71
**Drug likeness ^f^**	3.69	0.3	1.07	−1.2
**Molecular weight**	387.0	317.0	325.0	415.0
**Drug score ^g^**	0.74	0.71	0.39	0.17

^a^ Mutagenicity refers to the induction of permanent transmissible changes in the structure of the genetic material of cells or organisms; ^b^ Tumorigenicity refers to the process by which neoplastic cells are grown in tissue culture from tumors; ^c^ Irritant refers to a stimulus from compound which causes irritation; ^d^ Reproductive effective refers to adverse effect of compounds that interfere with the reproductive organs of an organism; ^e^ The logS value is estimated, and it is a unit-stripped logarithm (base 10) of the solubility measured in mol/liter; ^f^ It is a value of the compound’s drug-like properties. The drug likeness is calculated by summing up score values of those fragments that are present in the molecule under investigation. The fragment list was created by shredding 3300 traded drugs as well as 15,000 commercially available chemicals (Fluka), yielding a complete list of all available fragments; ^g^ Drug score is composed of drug likeness, cLogP, logS, molecular weight and factors of toxicity risk management, and is used to judge the compound’s overall potential to qualify for a drug candidate. The value of the drug score is between 0 and 1, and the larger value means better pharmacokinetic properties.

**Table 3:** Contribution of different parameters to the drug score of **M7594_0037** and its derivatives.

Parameters	M7594_0037	3AP-2	NA-2	M-2
**Score from cLoP**	0.947	0.987/1.0	0.998/1.0	0.986/1.0
**Score from logS**	0.716	0.917/1.0	0.965/1.0	0.822/1.0
**Score from molecular weight**	0.795/1.0	0.899/1.0	0.89/1.0	0.734/1.0
**Score from drug likeness**	0.975/1.0	0.575/1.0	0.744/1.0	0.23/1.0
**No risk of mutagenicity**	1.0/1.0	1.0/1.0	1.0/1.0	0.6/1.0
**No risk of tumorigenicity**	1.0/1.0	1.0/1.0	0.6/1.0	0.6/1.0
**Risk of irritating effect**	1.0/1.0	1.0/1.0	0.8/1.0	1.0/1.0
**No risk of reproductive effect**	1.0/1.0	1.0/1.0	1.0/1.0	1.0/1.0
**Average total drug score**	0.740	0.712	0.388	0.173
